# Inhibition of hypoxia-inducible factor 1α accumulation by glyceryl trinitrate and cyclic guanosine monophosphate

**DOI:** 10.1042/BSR20192345

**Published:** 2020-01-24

**Authors:** Judy Kim, Ivraym B. Barsoum, Harrison Loh, Jean-François Paré, D. Robert Siemens, Charles H. Graham

**Affiliations:** 1 Department of Biomedical and Molecular Sciences, Queen’s University, Kingston, Ontario, Canada; 2 Department of Urology, Queen’s University, Kingston, Ontario, Canada

**Keywords:** cGMP, hypoxia inducible factors, hypoxia, nitric oxide, nitroglycerin

## Abstract

A key mechanism mediating cellular adaptive responses to hypoxia involves the activity of hypoxia-inducible factor 1 (HIF-1), a transcription factor composed of HIF-1α, and HIF-1β subunits. The classical mechanism of regulation of HIF-1 activity involves destabilisation of HIF-1α via oxygen-dependent hydroxylation of proline residues and subsequent proteasomal degradation. Studies from our laboratory revealed that nitric oxide (NO)-mediated activation of cyclic guanosine monophosphate (cGMP) signalling inhibits the acquisition of hypoxia-induced malignant phenotypes in tumour cells. The present study aimed to elucidate a mechanism of HIF-1 regulation involving NO/cGMP signalling. Using human DU145 prostate cancer cells, we assessed the effect of the NO mimetic glyceryl trinitrate (GTN) and the cGMP analogue 8-Bromo-cGMP on hypoxic accumulation of HIF-1α. Concentrations of GTN known to primarily activate the NO/cGMP pathway (100 nM–1 µM) inhibited hypoxia-induced HIF-1α protein accumulation in a time-dependent manner. Incubation with 8-Bromo-cGMP (1 nM–10 µM) also attenuated HIF-1α accumulation, while levels of HIF-1α mRNA remained unaltered by exposure to GTN or 8-Bromo-cGMP. Furthermore, treatment of cells with the calpain (Ca^2+^-activated proteinase) inhibitor calpastatin attenuated the effects of GTN and 8-Bromo-cGMP on HIF-1α accumulation. However, since calpain activity was not affected by incubation of DU145 cells with various concentrations of GTN or 8-Bromo-cGMP (10 nM or 1 µM) under hypoxic or well-oxygenated conditions, it is unlikely that NO/cGMP signalling inhibits HIF-1α accumulation via regulation of calpain activity. These findings provide evidence for a role of NO/cGMP signalling in the regulation of HIF-1α, and hence HIF-1-mediated hypoxic responses, via a mechanism dependent on calpain.

## Introduction

Hypoxia-inducible factor 1 (HIF-1) is a key transcription factor that mediates cellular adaptations to hypoxia, including the acquisition of malignant properties in cancer cells. HIF-1 is a heterodimer composed of HIF-1α (120 kDa) and HIF-1β (91–94 kDa) subunits [1]. Together, the HIF-1 complex binds to hypoxia response elements (HREs) and transactivates target genes [2–5].

While HIF-1β is constitutively expressed, HIF-1α is regulated such that its protein levels determine HIF-1 transcriptional activity [2]. Under well-oxygenated conditions, prolyl residues 402 and 564 are hydroxylated by the prolyl-hydroxylase domain (PHD)-containing enzymes (PHD1, PHD2, and PHD3) [6]. This hydroxylation is required for the binding of the von Hippel-Lindau tumour suppressor protein (pVHL; the substrate recognition component of the E3 ubiquitin ligase complex), which leads to ubiquitylation and proteasomal degradation of HIF-1α [7]. Since oxygen is an absolute requirement for PHD enzyme activity, prolyl hydroxylation of HIF-1α is inhibited under hypoxic conditions, allowing HIF-1α to accumulate. While this is a well-characterised mechanism of regulation of HIF-1 activity, there is evidence that alternative pathways involving nitric oxide (NO) signalling regulate HIF-1 and, consequently, adaptations to hypoxia.

NO is produced endogenously as a product of the oxidation of l-arginine into l-citrulline in an oxygen-dependent reaction catalysed by the enzymes NO synthases (NOSs) [8]. NO plays key roles in the regulation of a vast array of biological functions as well as pathological states such as cancer. It has been shown that NO affects various aspects of cancer biology including cell proliferation, metastasis, angiogenesis, and resistance to therapy; however, the precise role of NO in tumour progression has been controversial, with studies suggesting either tumour-promoting effects [9–12] or tumour-inhibitory effects [13–16]. The apparent dichotomy of NO-mediated effects may be explained by the fact that NO can regulate phenotypes through a variety of mechanisms depending on the local concentration of NO and the molecular environment (e.g. redox status) [17]. At high concentrations (>1 µM), NO can undergo reactions with oxygen or superoxide radicals to produce reactive oxygen species that alter protein function via nitrosylation and nitration [8,18]. At low concentrations (<1 µM), NO can interact with transition metals, such as iron in haem proteins [8,18]. The haem-containing enzyme soluble guanylyl cyclase (sGC) is the main target of NO that mediates most of its downstream effects by catalysing the conversion of guanosine triphosphate (GTP) into cyclic guanosine monophosphate (cGMP) [8]. cGMP subsequently activates various downstream effectors, of which cGMP-dependent protein kinase (PKG) is responsible for many of the effects of cGMP via phosphorylation of molecules that regulate gene expression and cell function [8,17].

Previous studies have shown regulatory effects of NO on HIF-1α accumulation and HIF-1 activity, all of which have been attributed to cGMP-independent mechanisms [19–26]. Proposed mechanisms of NO-mediated inhibition of HIF-1*α* accumulation in hypoxia include inhibition of mitochondrial activity leading to increased O_2_ availability for re-activation of PHD enzymes [20,27], increased availability of iron and activation of PHDs via mechanisms involving reactive nitrogen species [24,25], direct activation of PHDs [28], and induction of PHD expression [22]. Zhou et al. [26] revealed that an alternative, PHD/pVHL/proteasome-independent mechanism involving the calpain (Ca^2+^-activated protease) system mediates NO-induced HIF-1α degradation. That study, however, did not investigate a role of cGMP in HIF-1*α* degradation.

Studies from our laboratory demonstrated that the acquisition of hypoxia-induced malignant properties in tumour cells, such as invasiveness, metastatic ability, and drug resistance, is inhibited by activation of the low concentration NO/cGMP signalling pathway, whereas inhibition of this pathway in well-oxygenated cells results in phenotypes similar to those induced by hypoxia [4,5,17,29–34]. Given the central role of HIF-1 in mediating these hypoxic responses, in the present study, we tested the hypothesis that HIF-1α is a downstream target of the NO/cGMP signalling pathway and, therefore, an important component of the mechanism by which NO modulates hypoxia-induced phenotypes.

## Materials and methods

### Cells and culture conditions

The human prostate carcinoma cell line DU145 was obtained from the American Type Culture Collection (ATCC; Manassas, VA, U.S.A.). Cells were maintained in RPMI 1640 medium (Life Technologies Invitrogen Corporation, Burlington, ON, Canada) supplemented with 5% fetal bovine serum (Sigma–Aldrich Canada Ltd., Oakville, ON, Canada) and plated in six-well plates at 60–70% confluence (to avoid pericellular hypoxia resulting from high-density cultures [35]) at the start of all experiments. Following a 24-h incubation in standard culture conditions (20% O_2_), the culture medium was changed and the cultures were incubated in 20% O_2_ or hypoxia (0.2% O_2_). For incubations in standard conditions (20% O_2_), cells were placed in a Thermo Forma CO_2_ incubator (5% CO_2_ in air at 37°C) whereas for incubations in hypoxia, cells were placed in airtight chambers that were flushed with a gas mixture of 5% CO_2_/95% N_2_ (BOC, Kingston, ON, Canada) and maintained at 37°C. Oxygen concentrations within these chambers were kept at 0.2% using Pro-Ox model 110 O_2_ regulators (Biospherix, Redfield, NY, U.S.A.).

To assess dose-dependent effects of NO/cGMP signalling on hypoxia-induced HIF-1α accumulation, randomly selected cultures were incubated with various concentrations (1 nM–10 µM) of glyceryl trinitrate (GTN) (Omega Laboratories Ltd., Montreal, QC, Canada) or 8-Bromo-cGMP (Sigma–Aldrich Canada) administered at the beginning of the 4-h incubation in standard or hypoxic conditions. Subsequently, temporal effects of NO/cGMP signalling were determined by exposing cells to 1 µM GTN or 8-Bromo-cGMP for 4, 8, 16, and 24 h in 20% O_2_ or 0.2% O_2_.

The involvement of calpain in the NO/cGMP-mediated regulation of HIF-1α was analysed by incubating cells with GTN (1 µM for 4 h) or 8-Bromo-cGMP (1 µM for 4 h), or in combination with the selective calpain inhibitor calpastatin (CS) peptide (2 µM for 4 h; Calbiochem/EMD Biosciences, San Diego, CA, U.S.A.) in 20% O_2_ or 0.2% O_2_. The concentration of CS used in the present study was previously shown to effectively inhibit calpain-mediated degradation of HIF-1α [26].

### Determination of HIF-1*α* protein accumulation

Following incubation under various conditions, cells were frozen immediately by rapidly discarding the medium and placing the culture plates in liquid nitrogen. Cells were lysed with a buffer containing 2% SDS, 10 mM Tris, 0.15 M NaCl (pH 7.6), and Complete Protease Inhibitor Cocktail (Roche Diagnostics Canada, Laval, QC, Canada). Lysates of equal protein concentrations were resolved on 7.5% SDS/polyacrylamide Next Gels (Amresco/Cedarlane Laboratories, Burlington, ON, Canada) and transferred to Immobilon-P membranes (Millipore Corporation, Bedford, MA, U.S.A.) via a wet-transfer system (Bio-Rad Laboratories). Western immunoblots were performed on the membranes using mouse monoclonal anti-human HIF-1α antibody (1:250 dilution, BD Biosciences, Mississauga, ON, Canada) and mouse monoclonal anti-β-actin antibody (1:5000 dilution, Sigma–Aldrich Canada) followed by incubation with HRP-conjugated goat anti-mouse IgG secondary antibody (1:5000 in 1% milk/TBS; Bio-Rad Laboratories) and detection by enhanced chemiluminescence (PerkinElmer Life Sciences Inc., Boston, MA, U.S.A.) and exposure to Kodak X-Omat film (Eastman Kodak, Rochester, NY, U.S.A.).

### Assessment of HIF-1*α* mRNA levels

Total RNA was isolated using Total RNA Mini Kit (Geneaid Biotech Ltd./Frogga Bio, Toronto, ON, Canada) according to the manufacturer’s protocol. One microgram of total RNA was reverse-transcribed using random hexamers (100 µM; Cortec DNA Service Laboratories Inc., Kingston, ON, Canada) and Omniscript RT Kit (Qiagen Inc., Toronto, ON, Canada) according to the manufacturer’s guidelines. The resultant cDNAs were used as templates for real-time PCR amplification using a LightCycler 480 (Roche) and the KAPA SYBR FAST Master Mix (Kapa Biosystems/D-Mark Biosciences, Toronto, ON, Canada).

Primer sets were designed from published NIH Genbank mRNA sequences (NIH Centre for Biotechnical Information, Bethesda, MD, U.S.A.) using Primer Design 2.01 software (Scientific & Educational Software, Cary, NC, U.S.A.). The primer sequences were as follows: *HIF-1α*, forward 5′-CGACACAGCCTGGATATGAA-3′ and reverse 5′- TCCTGTGGTGACTTGTCCTT-3′ (T_a_ = 63°C, 200 nM; Eurofins mwg/operon, Huntsville, AL, U.S.A.); β-actin, forward 5′-CTGGACTTCGAGCAAGAGAT-3′ and reverse 5′-GATGTCCACGTCACACTTCA-3′ (T_a_ = 63°C, 200 nM; Euronfins mwg/operon). The cycling conditions consisted of a denaturation step (95°C for 5 min) followed by 40 cycles at 95°C for 15 s, 63°C for 20 s, and 72°C for 5 s. Levels of gene expression were calculated using the standard curve method for each gene. The primers had an efficiency range between 1.7 and 2.0.

### Calpain activity assay

Calpain activity was determined using a calpain activity assay kit (catalogue# MAK228, Sigma–Aldrich) in which fluorescence emitted by the calpain substrate Ac-LLY-AFC was assessed. Cells incubated in 20% O_2_ or 0.2% O_2_ for 24 h were flash-frozen and whole lysates were extracted using the buffer provided in the kit. Eighty-five micrograms of extracted proteins were incubated with calpain substrate at 37°C for 1 h.

Fluorescence was measured using a Spectramax iD3 (Molecular Devices, San Jose, CA, U.S.A.) plate reader with the excitation filter set at 400 nm and the emission filter set at 505 nm. Cell extracts incubated with the calpain inhibitor Z-LLY-FMK included with the kit were used as negative controls to establish basal fluorescence.

### Calculations and statistical analysis

To quantify HIF-1α protein levels from Western blot experiments, x-ray films were scanned and densitometric analysis was performed using Image Processing and Analysis in Java (ImageJ, National Institute of Mental Health, Bethesda, Maryland, U.S.A.). The relative levels of HIF-1α protein and mRNA were determined by densitometry after calculating the HIF-1α/β-actin ratio to account for differences in sample loading.

Results are presented as means ± the standard error of the mean (SEM). All statistical analyses were performed using Prism 6.0 Software (GraphPad Software Inc., La Jolla, CA, U.S.A.). Based on the experimental design, statistical significance was determined using one-way analysis of variance (ANOVA) or two-way repeated-measures ANOVA followed by Bonferroni’s *post hoc* test when comparisons involved three or more groups. A two-tailed paired Student’s *t* test was used to statistically analyse the effect of hypoxia on calpain activity. Differences in mean values were considered statistically significant at *P*<0.05.

## Results

### Effect of GTN on HIF-1α expression

**Figure 1:**
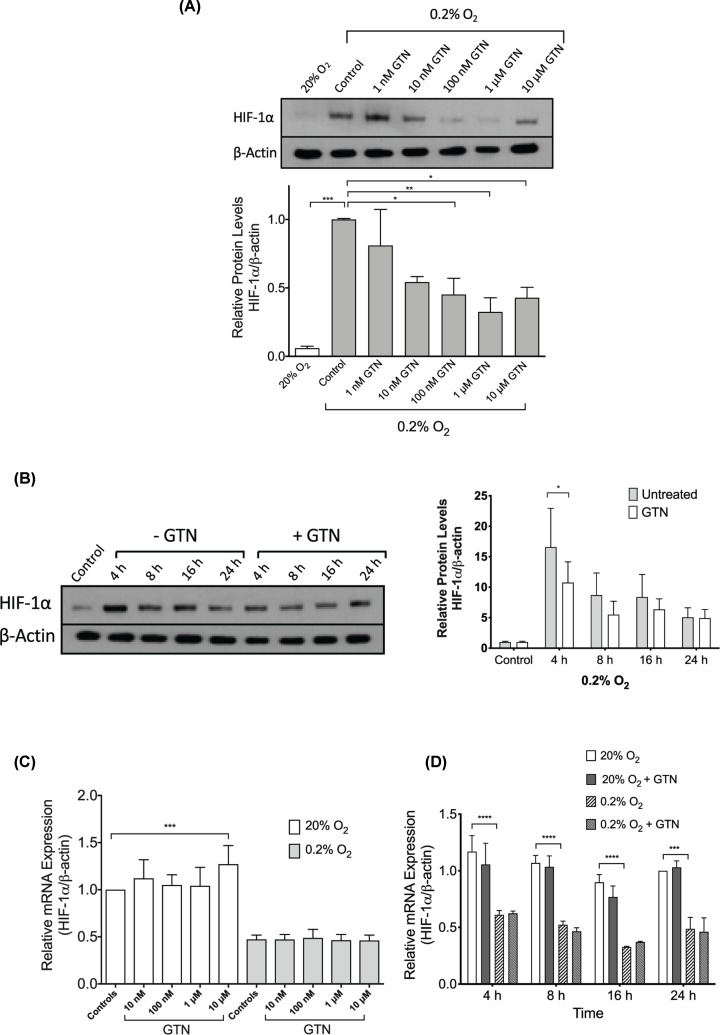
Effect of GTN on HIF-1α expression in DU145 cells (**A**) Western blot analysis of HIF-1α protein levels in cells cultured for 4 h in 20% O_2_ or 0.2% O_2_ in the presence or absence of various concentrations of GTN (1 nM–10 µM). Data are from two independent experiments. (**B**) Western blot analysis of HIF-1α protein in cells cultured for various durations (4–24 h) in 0.2% O_2_ in the presence or absence of GTN (1 µM). Control cells were incubated for 24 h in 20% O_2_. Data were obtained from four independent experiments. (**C**) Quantitative reverse transcriptase polymerase chain reaction (qRT-PCR) analysis of HIF-1α expression in cells exposed to 20% O_2_ or 0.2% O_2_ for 24 h in the presence or absence of various concentrations of GTN (10 nM–10 µM). Data were obtained from three independent experiments. (**D**) qRT-PCR analysis of HIF-1α mRNA in cells incubated in 20% O_2_ or 0.2% O_2_ for various periods of time (4–24 h) in the presence or absence of GTN (1 µM). Data are from three independent experiments. Bars in (A–D) represent mean ± SEM. *, *P*<0.05; **, *P*<0.01; ***, *P*<0.001; ****, *P*<0.0001, one-way ANOVA (A,C) or two-way repeated measures ANOVA (B,D) followed by Bonferroni’s post hoc test.

Immunoblot analysis revealed that incubation of cells in 0.2% O_2_ for 4 h resulted in a significant increase in HIF-1α protein levels compared with cells incubated in 20% O_2_, while administration of GTN (1 nM–10 µM) at the onset of the 4-h hypoxic exposure prevented the accumulation of HIF-1α in a concentration-dependent manner (Figure 1A). A significant inhibitory effect of GTN on HIF-1α accumulation was observed at concentrations of 100 nM, 1 µM, and 10 µM.

Further analysis revealed that the inhibitory effect of GTN (1 µM) on hypoxia-induced HIF-1α accumulation was most robust at the 4-h time point (Figure 1B). GTN did not significantly alter HIF-1α protein levels in cells incubated in 20% O_2_ at all time points examined (Supplementary Figure S1).

In contrast with the hypoxic induction of HIF-1α protein accumulation, exposure of DU145 cells to 0.2% O_2_ for 24 h resulted in a significant *decrease* in HIF-1α mRNA levels compared with cells incubated in 20% O_2_ (Figure 1C). Administration of GTN did not affect HIF-1α mRNA expression at all concentrations (10 nM–10 µM) tested (Figure 1C). Similarly, time-course analysis revealed that various durations of hypoxic exposure (4–24 h) significantly decreased HIF-1α mRNA levels and addition of GTN (1 µM) did not alter HIF-1α mRNA expression in either 20% O_2_ or 0.2% O_2_ at all time points examined (Figure 1D).

### Effect of 8-Bromo-cGMP on HIF-1α accumulation

**Figure 2:**
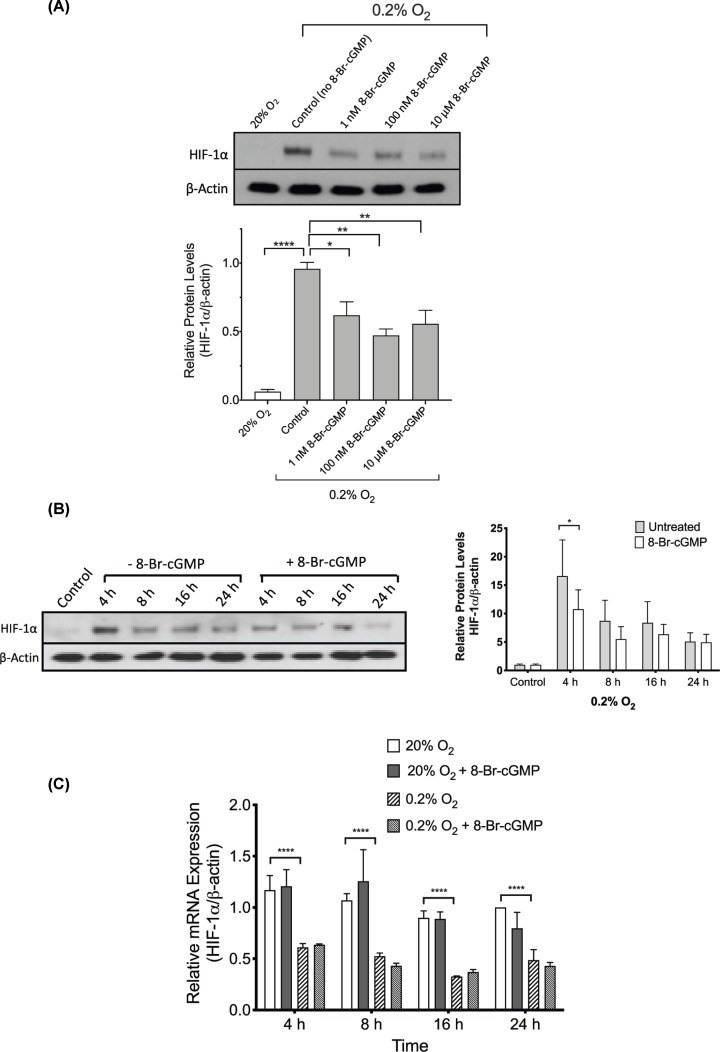
Effect of 8-Bromo-cGMP on HIF-1α expression in DU145 cells (**A**) Western blot analysis of HIF-1α protein levels in cells cultured for 4 h in 20% O_2_ or 0.2% O_2_ in the presence or absence of various concentrations of 8-Br-cGMP (1 nM–10 µM). Data are from three independent experiments. (**B**) Western blot analysis of HIF-1α protein in cells cultured for various durations (4–24 h) in 0.2% O_2_ in the presence or absence of 8-Bromo-cGMP (8-Br-cGMP, 1 µM). Control cells were incubated for 24 h in 20% O_2_. Data are from three independent experiments. Quantitative analysis of relative levels of protein is shown in the graph on the right. (**C**) qRT-PCR analysis of HIF-1α mRNA in cells incubated in 20% O_2_ or 0.2% O_2_ for various periods of time (4–24 h) in the presence or absence of 8-Br-cGMP (1 µM). Data are from three independent experiments. Bars in (A–C) represent mean ± SEM. *, *P*<0.05; **, *P*<0.01; ****, *P*<0.0001, one-way ANOVA (A) or two-way repeated measures ANOVA (B,C) followed by Bonferroni’s post hoc test.

Incubation of cells with the non-hydrolysable cGMP analogue 8-Bromo-cGMP (1 nM, 100 nM, and 10 µM) for 4 h significantly inhibited the hypoxic accumulation of HIF-1α protein at all concentrations of the cGMP analogue used (Figure 2A,B). The significant effect of 8-Bromo-cGMP on HIF-1*α* accumulation was only observed at 4-h incubation in hypoxia (Figure 2B). HIF-1α protein levels in cells incubated in 20% O_2_ were unaffected by exposure to 8-Bromo-cGMP at all time points examined (Supplementary Figure S2).

Exposure of DU145 cells to 8-Bromo-cGMP (1 µM) in 20% O_2_ or 0.2% O_2_ for various time periods (4–24 h) did not result in a significant change in HIF-1α mRNA levels compared with controls (cells incubated without 8-Bromo-cGMP) at all time points tested, as determined by qRT-PCR (Figure 2C).

### Calpain and NO/cGMP-mediated inhibition of HIF-1α protein accumulation

**Figure 3:**
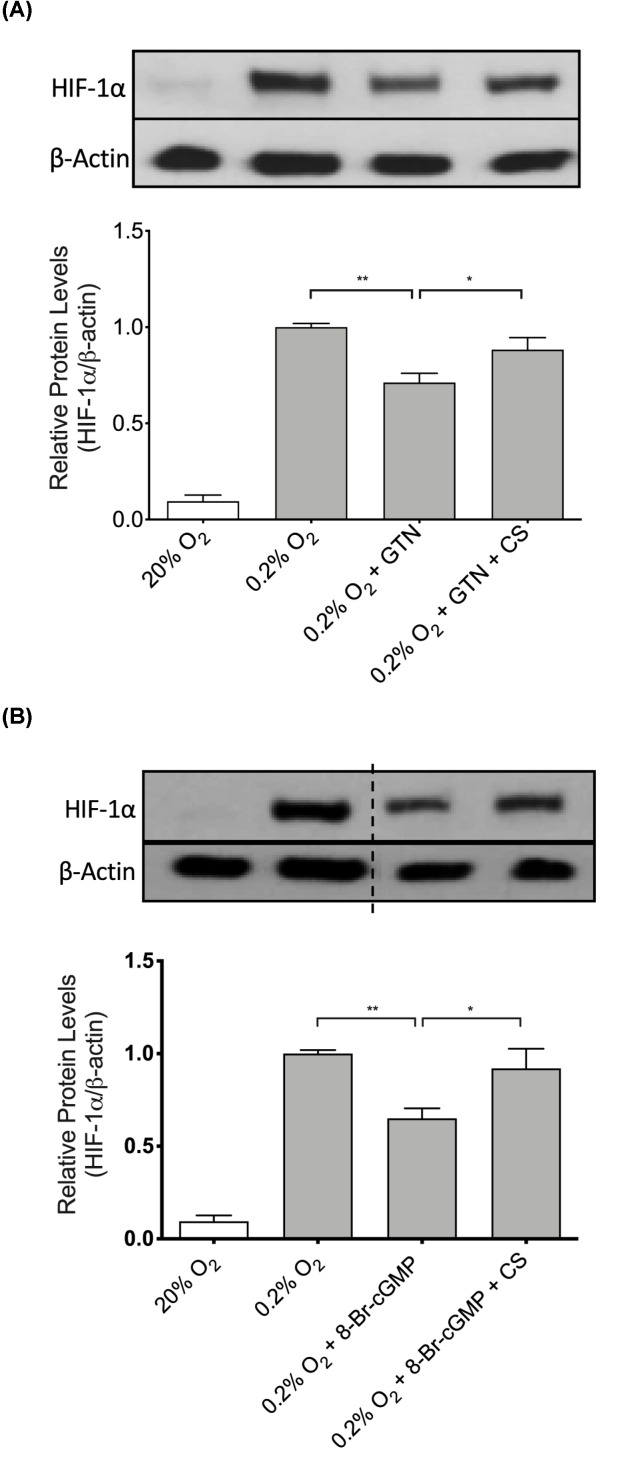
Effect of calpastatin on hypoxia-induced HIF-1α accumulation in the presence of NO mimetics Western blot analysis of HIF-1α protein levels in cells incubated for 4 h in 20% O_2_ or 0.2% O_2_ in the presence or absence of GTN (1 µM; (**A**) or 8-Bromo-cGMP (8-Br-cGMP, 1 µM; (**B**), either alone or in combination with the peptide calpain inhibitor calpastatin (CS; 2 µM) (*n*= 4 for both (A,B)). The separating vertical dotted line in (B) indicates where the image was cut and reordered to facilitate description of the data. Bars in both (A,B) represent mean ± SEM. *, *P*<0.05; **, *P*<0.01, one-way ANOVA followed by Bonferroni’s post hoc test.

**Figure 4:**
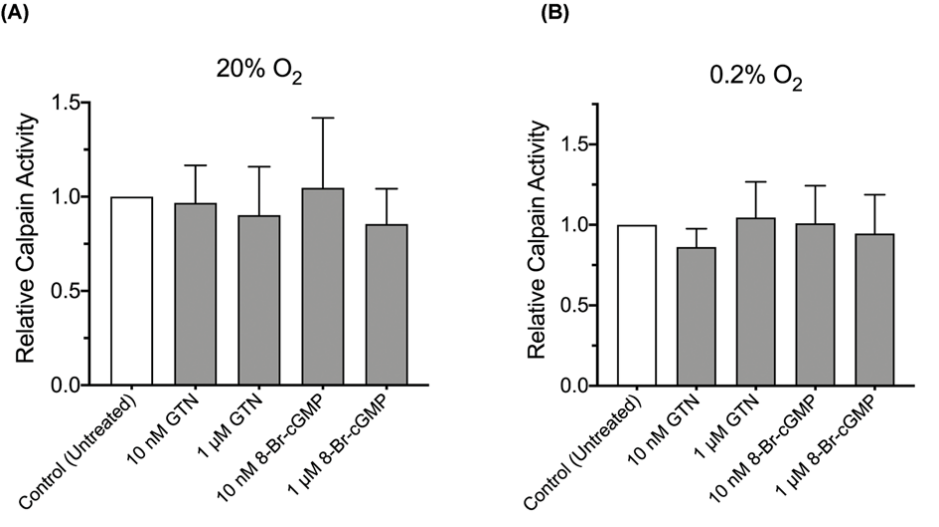
Effect of culture conditions on calpain activity in DU145 cells Compared with controls, there were no significant differences in calpain activity in cells incubated with GTN (10 nM or 1 µM) or 8-Bromo-cGMP (8-Br-cGMP; 10 nM or 1 µM) in 20% O_2_ (**A**) or 0.2% O_2_ (**B**). Bars represent the means ± SEM of six to ten independent experiments.

To determine the role of calpain in the NO/cGMP-mediated attenuation of HIF-1α accumulation, DU145 cells were treated with GTN (1 µM) or 8-Bromo-cGMP (1 µM) alone, or in combination with the specific calpain inhibitor CS (2 µM) at the onset of a 4-h incubation in 0.2% O_2_. While GTN alone reduced HIF-1α protein accumulation in cells exposed to hypoxia, co-incubation with CS significantly attenuated the inhibitory effect of GTN on HIF-1α accumulation (Figure 3A). Similarly, 8-Bromo-cGMP alone significantly reduced hypoxic accumulation of HIF-1α protein while addition of CS significantly blocked the effect of 8-Bromo-cGMP in DU145 cells (Figure 3B). Finally, results of the calpain activity assay revealed that incubation of DU145 cells with various concentrations of GTN or 8-Bromo-cGMP (10 nM or 1 µM) in 20% O_2_ or 0.2% O_2_ did not affect calpain activity (Figure 4A,B), and there were no significant differences in mean calpain activity levels in cells incubated in 20% O_2_ versus 0.2% O_2_ (*P*=0.24).

## Discussion

The main finding of the present study was that GTN and 8-Bromo-cGMP inhibited the hypoxia-mediated accumulation of HIF-1*α* via a mechanism that likely involves calpain activity. While the role of calpain in the regulation of HIF-1*α* accumulation by NO was previously reported [26], our study reveals, for the first time, that cGMP likely mediates this effect of NO. This finding is important because most studies on the regulation of HIF-1*α* by NO have primarily centred on cGMP-independent, high concentration effects of NO. A well-known mechanism of HIF-1*α* regulation involves PHD-mediated hydroxylation of prolyl residues (Pro^402^ and Pro^564^ in human HIF-1*α*) in the oxygen degradation domain of HIF-1*α* in an oxygen-dependent manner. Our study provides evidence that NO signalling via cGMP generation and inhibition of calpain activity is an additional mechanism of HIF-1 regulation.

Both concentration and duration of exposure to NO are critical determinants of the quality and magnitude of the biological response to exogenously administered NO mimetics [36]. Analysis of the effect of GTN on HIF-1α accumulation at various concentrations and exposure times revealed that relatively low concentrations (100 nM and 1 µM) of GTN were able to significantly attenuate hypoxic accumulation of HIF-1α protein and that the inhibitory effect of GTN was rapid and possibly transient, peaking at 4-h treatment. Most of the low concentration effects of NO are attributable to the activation of the NO/cGMP signalling pathway in which NO binds to sGC and subsequently induces cGMP production and activation of downstream effectors [8]. Thus, the observed inhibitory effect of GTN, at such low concentrations as 100 nM and 1 µM, on hypoxia-induced HIF-1α accumulation suggests that this effect occurs via activation of the cGMP-dependent signalling pathway. This is further supported by results of previous studies showing that levels of NO in cells treated with ≤1 µM of NO donors, including GTN, were undetectable using standard assays that measure nitrate and nitrite formation as an index for NO production [30]; this indicated that NO levels were lower than those required to produce the reactive nitrogen species nitrate/nitrite and, as such, exerted their effects predominantly through the low-concentration NO/cGMP pathway [30].

Based on the observed effect of GTN on HIF-1α accumulation and given the central role of HIF-1α in mediating hypoxic responses, it is possible that the inhibitory effects of low concentrations of GTN on hypoxia-induced phenotypes observed in previous studies [5,29–34] are, in part, a result of interfering with HIF-1α accumulation. Interestingly, while induction and attenuation of these previously reported phenotypes (i.e. increase in invasion and metastasis, drug resistance, and immune escape) by hypoxia and by low concentrations of GTN, respectively, were evident following 24-h exposures, a similar pattern of effect of hypoxia and GTN on HIF-1α protein accumulation was observed as early as, and most prominently, 4 h. This may perhaps reflect the time it takes to effect a change in transcription and protein expression to finally manifest phenotypic alterations, once HIF-1α accumulation and, consequently, HIF-1 activity are modified.

In addition to the results showing that GTN, at a concentration known to primarily activate the cGMP-dependent pathway, inhibited hypoxic accumulation of HIF-1α, evidence in support of the participation of the cGMP signalling pathway in HIF-1α regulation was provided by the finding that 8-Bromo-cGMP (i.e. a cGMP analogue) similarly attenuated hypoxia-induced HIF-1α accumulation. These results are in agreement with previous studies [30–32] showing that NO via cGMP production prevents hypoxia-mediated acquisition of malignant phenotypes in tumour cells and suggest that modulation of HIF-1α may be an important aspect of the mechanism by which NO/cGMP signalling regulates hypoxic responses. Although many of the studies examining the regulatory effects of NO on HIF-1α accumulation and/or HIF-1 activity have proposed cGMP-independent mechanisms of HIF-1 regulation, Tsuruda et al. [37] have found that activation of sGC/cGMP signalling in cultured cardiomyocytes decreased hypoxia-induced HIF-1α protein accumulation; this further supports the notion that the cGMPdependent signalling interferes with hypoxic induction of HIF-1α accumulation and suggests that such mechanism of HIF-1α modulation may apply to a variety of normal and transformed cells.

Interestingly, in contrast with its effects on HIF-1α accumulation in hypoxia, neither GTN nor 8-Bromo-cGMP altered HIF-1α protein levels in DU145 cells under well-oxygenated conditions (20% O_2_) (Supplementary Figures S1 and S2). This selective action of GTN and 8-Br-cGMP is in line with previous studies showing that such activation of NO/cGMP signalling inhibited malignant phenotypes of hypoxic tumour cells without affecting well-oxygenated cells [29,30,32,33], highlighting its potential to selectively target the more malignant hypoxic cells. This phenomenon may be explained by the fact that endogenous NO production requires O_2_ [8] and that exposure to low O_2_ conditions limits cellular NO synthesis [38,39] as well as cGMP production [31,40,41]. Consequently, activation of the NO/cGMP signalling likely targets hypoxic cells (i.e. cells with impaired NO production and signalling) with little or no effect on oxygenated cells (i.e. cells with normal NO production and signalling).

The present study revealed that GTN or 8-Bromo-cGMP did not alter the levels of HIF-1α mRNA in either hypoxic (0.2% O_2_) or oxygenated (20% O_2_) conditions, suggesting that the NO/cGMP-mediated attenuation of hypoxia-induced HIF-1α accumulation occurs via translational or post-translational mechanisms. It has been reported that NO decreases HIF-1α protein abundance and hence HIF-1 activity via a PHD/pVHL/proteasome-independent mechanism that involves calpain (Ca^2+^-activated protease)-mediated degradation of HIF-1α [26]; however, that report did not address the role of cGMP signalling in the proposed mechanism of HIF-1α regulation. In accordance with and extending those findings, the results of the present study indicate that the NO/cGMP-induced inhibition of HIF-1α accumulation likely requires calpain activity.

Cyclic GMP has been shown to increase intracellular levels of Ca^2+^ [42–44], the primary activator of calpain, and this has been linked to increases in calpain activity [45]. Furthermore, it has been found that the downstream effector of cGMP, PKG, is required for NO/cGMP-mediated generation of the Ca^2+^ signal and activation of µ-calpain [46]. In our study, regardless of oxygenation levels, we did not observe changes in calpain activity in DU145 cells incubated in the presence of GTN or 8-Bromo-cGMP. These findings indicate that, while the presence of calpain may be critical for the observed effect of NO/cGMP signalling, the decreased accumulation of HIF-1α in hypoxic cells incubated with NO mimetics is not likely due to increased calpain activity. Thus, the precise mechanism of NO-mediated inhibition of HIF-1α accumulation requires further investigation. PHDs are also subject to regulation by Ca^2+^ and it has been shown that chelation of intracellular Ca^2+^ induces HIF-1α accumulation and HIF-1 transactivation by inhibiting PHD activity under oxygenated conditions [47]. Based on the results presented in this study, the potential role of PHD/pVHL/proteasome pathway in the NO/cGMP-induced attenuation of HIF-1α accumulation cannot be excluded and further studies are needed to determine whether PHDs and calpains synergistically mediate HIF-1α degradation. In addition to HIF-1α, calpain has been found to mediate the degradation of HIF-2α [48], suggesting that HIFs are among the many calpain substrates and that calpain plays an important role in the regulation of HIFs and hence hypoxic responses.

Overall, the findings presented in the present study provide new mechanistic insights into the NO-mediated regulation of HIF-1α accumulation, and support the concept that tumour hypoxic responses involving HIF-1 activity may be prevented by activation of the NO/cGMP signalling pathway. The proposed pathway of HIF-1α modulation may have physiological relevance not only in cancer but also in other pathological conditions as well as normal biological processes characterized by low oxygen levels.

## Supplementary Material

Supplementary Figures S1 and S2

## Abbreviations

ANOVA: analysis of variance
cGMP: cyclic guanosine monophosphate
CS: calpastatin
GTN: glyceryl trinitrate
HIF-1: hypoxia-inducible factor 1
HRP: horse radish peroxidase
NO: nitric oxide
PHD: prolyl-hydroxylase domain
PKG: cGMP-dependent protein kinase
pVHL: von Hippel-Lindau tumour suppressor protein
SEM: standard error of the mean
sGC: soluble guanylyl cyclase
