# Supplementary material for: Inhibition of hypoxia-inducible factor 1α accumulation by glyceryl trinitrate and cyclic guanosine monophosphate
Source: Biosci Rep. 2020 Jan 24;40(1):BSR20192345. doi: 10.1042/BSR20192345 (PMC6981098; doi:10.1042/BSR20192345)
Supplement: Supplementary Figures S1 and S2 [file BSR-2019-2345_supp.pdf]

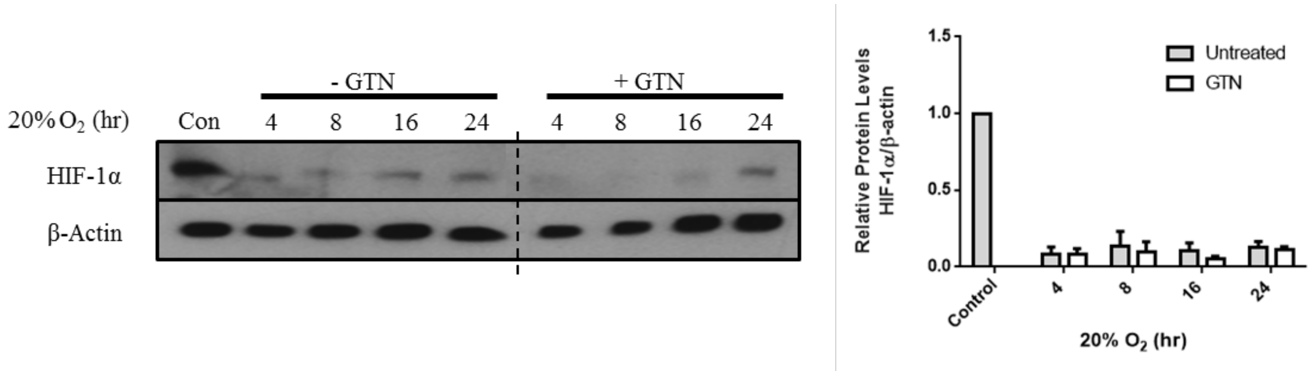

Supplementary Figure 1. Effect of GTN on HIF-1α protein levels in DU145 cells under well-oxygenated conditions

Representative blot and quantification of HIF-1α protein in cells incubated in 20% O_2 for various times (4-24 hours) in the presence or absence of 1 μM GTN are shown (n=3). Control (Con) cells were incubated for 24 hours in 0.2% O_2. The separating vertical dotted line indicates where the image was cut and reordered to facilitate description of the data. Bars represent mean ± SEM; two-way repeated measures ANOVA followed by Bonferroni post hoc test.

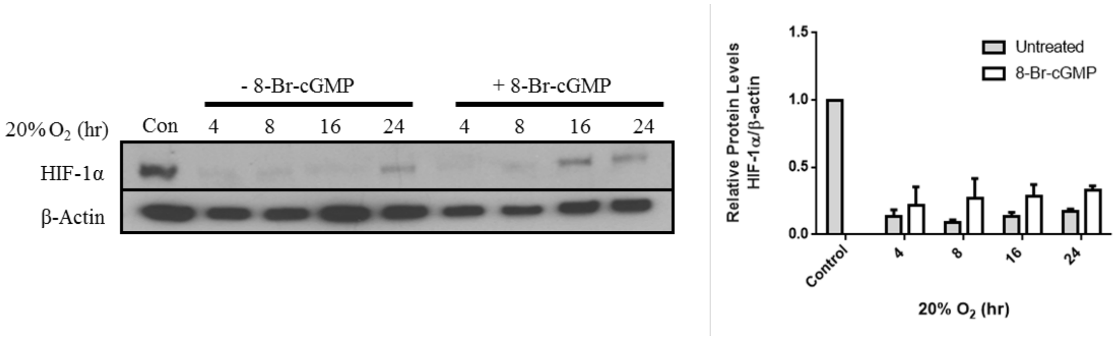

Supplementary Figure 2. Effect of 8-Br-cGMP on HIF-1α protein levels in DU145 cells under well-oxygenated conditions

Representative blot and quantification of HIF-1α protein in cells incubated in 20% O_2 for various periods of time (4-24 hours) in the presence or absence of 1 μM 8-Bromo-cGMP are presented (n=3). Control (Con) cells were incubated for 24 hours in 0.2% O_2. Bars represent mean ± SEM; two-way repeated measures ANOVA followed by Bonferroni post hoc test.
